# Comment on “The Crucial Role of Physical Activity Index in Predicting the Incidence of Pacemaker Syndrome”

**DOI:** 10.1002/joa3.70261

**Published:** 2026-01-02

**Authors:** Ahmet Yılmaz

**Affiliations:** ^1^ Department of Cardiology Karaman Training and Research Hospital Karaman Türkiye

To the Editor,

I read with great interest the article by Malekrah et al., entitled “The Crucial Role of Physical Activity Index in Predicting the Incidence of Pacemaker Syndrome,” published in the Journal of Arrhythmia. The study makes a noteworthy contribution by addressing the potential role of physical activity in the pathogenesis of pacemaker syndrome (PMS). However, several important limitations in methodological design and parameter selection restrict the clinical generalizability of the findings.

First, implanting dual‐chamber devices in all patients and reprogramming them to VVIR mode at discharge does not reflect clinical practice. Large randomized trials (PASE, CTOPP, MOST) have typically performed mode changes only in symptomatic cases or for comparative analysis [1, 2, 3]. Routinely implanting dual‐chamber devices but programming them in single‐chamber mode lacks clinical justification and creates a non‐physiological environment of AV dyssynchrony. Therefore, the reported incidence of PMS was obtained under a protocol different from real‐life practice and should be interpreted with caution.

Second, the study population being limited to individuals under 65 years of age, with left ventricular ejection fraction (LVEF) ≥ 50% and sinus rhythm, excludes the highest‐risk groups for PMS. The current ESC Guidelines on Cardiac Pacing and CRT [4] and the HRS/APHRS/LAHRS Guideline on Cardiac Physiologic Pacing [5] emphasize that hemodynamic intolerance related to PMS is more pronounced in patients with low EF and atrial fibrillation (AF). The exclusion of these patients limits the applicability of the results to a narrow group of “young, active, normal EF, and sinus rhythm” individuals.

Third, many key parameters essential for understanding PMS pathophysiology were not evaluated in this study. Recent PMS studies have focused on hemodynamic indicators (blood pressure, stroke volume, cardiac output), electrophysiological variables (% ventricular pacing, AV interval, retrograde VA conduction time), and particularly mechanical synchrony parameters (global longitudinal strain, intraventricular dyssynchrony, mitral inflow patterns) [6, 7, 8, 9]. None of these parameters were reported in the work of Malekrah et al.

Fourth, the study demonstrated an increased incidence of PMS among individuals with high levels of physical activity. However, this finding may reflect increased symptom awareness or greater perception of effort‐related symptoms rather than a causal relationship. Physical activity level was assessed using the self‐reported IPAQ questionnaire, which carries a risk of measurement bias. Moreover, the study did not report whether higher activity actually led to greater ventricular pacing rates since parameters such as true pacing burden (%V‐pacing), AV interval, and rate‐adaptive sensor settings were not provided; the hemodynamic mechanism remains unsupported.

In conclusion, the study attempts to explain the multifactorial nature of PMS through a single variable‐the physical activity index. A model that does not simultaneously evaluate mechanical, electrophysiological, and clinical parameters cannot accurately represent the true pathophysiology of PMS. Future studies should include patients with low EF, a broader age range, and various pacing modes; an analytical approach supported by objective exercise testing and advanced echocardiographic measurements will enhance the scientific clarity of this issue.

Sincerely,

## Conflicts of Interest

The author declares no conflicts of interest.
