# Correction to ‘Comment on “The Crucial Role of Physical Activity Index in Predicting the Incidence of Pacemaker Syndrome”’

**DOI:** 10.1002/joa3.70272

**Published:** 2026-02-02

A. Yılmaz, “Comment on “The Crucial Role of Physical Activity Index in Predicting the Incidence of Pacemaker Syndrome”,” *Journal of Arrhythmia* 42, no. 1 (2026): e70261, https://doi.org/10.1002/joa3.70261.

In the published article, References 1, 2, 3, 4, and 6 contained errors. The corrected references are provided below.

**The corrected references are as follows:**

**Citation 1:** G. A. Lamas, E. J. Orav, B. S. Stambler, K. A. Ellenbogen, E. B. Sgarbossa, S. K. S. Huang, and B. L. Wilkoff, “Quality of Life and Clinical Outcomes in Elderly Patients Treated With Ventricular Pacing as Compared With Dual‐Chamber Pacing,” *New England Journal of Medicine* 338, no. 16 (1998): 1097–1104.

**Citation 2:** S. J. Connolly, C. R. Kerr, M. Gent, R. S. Roberts, S. Yusuf, A. M. Gillis, and D. M. Newman, “Effects of Physiologic Pacing Versus Ventricular Pacing on the Risk of Stroke and Death due to Cardiovascular Causes,” *New England Journal of Medicine* 342, no. 19 (2000): 1385–1391.

**Citation 3:** M. S. Link, A. S. Hellkamp, N. M. Estes, E. J. Orav, K. A. Ellenbogen, B. Ibrahim, and MOST Study Investigators, “High Incidence of Pacemaker Syndrome in Patients With Sinus Node Dysfunction Treated With Ventricular‐Based Pacing in the Mode Selection Trial (MOST),” *Journal of the American College of Cardiology* 43, no. 11 (2004): 2066–2071.

**Citation 4:** M. Glikson, J. C. Nielsen, M. B. Kronborg, Y. Michowitz, A. Auricchio, I. M. Barbash, and K. K. Witte, “2021 ESC Guidelines on Cardiac Pacing and Cardiac Resynchronization Therapy: Developed by the Task Force on Cardiac Pacing and Cardiac Resynchronization Therapy of the European Society of Cardiology (ESC) With the Special Contribution of the European Heart Rhythm Association (EHRA),” *EP Europace* 24, no. 1 (2022): 71–164.

**Citation 6:** V. Somma, F. J. Ha, S. Palmer, U. Mohamed, and S. Agarwal, “Pacing‐Induced Cardiomyopathy: A Systematic Review and Meta‐Analysis of Definition, Prevalence, Risk Factors, and Management,” *Heart Rhythm* 20, no. 2 (2023): 282–290.

We apologize for this error.

